# The dual-path hypothesis for the emergence of anosognosia in Alzheimer’s disease

**DOI:** 10.3389/fneur.2023.1239057

**Published:** 2023-11-02

**Authors:** Katia Andrade, Thomas Guieysse, Takfarinas Medani, Etienne Koechlin, Dimitrios Pantazis, Bruno Dubois

**Affiliations:** ^1^Institute of Memory and Alzheimer’s Disease (IM2A), Department of Neurology, Assistance Publique-Hôpitaux de Paris (AP-HP), Sorbonne University, Pitié-Salpêtrière Hospital, Paris, France; ^2^Frontlab, Paris Brain Institute (Institut du Cerveau, ICM), AP-HP, Pitié-Salpêtrière Hospital, Paris, France; ^3^Signal and Image Processing Institute, University of Southern California, Los Angeles, CA, United States; ^4^École Normale Supérieure, Laboratoire de Neurosciences Cognitives et Computationnelles, Paris, France; ^5^McGovern Institute for Brain Research, Massachusetts Institute of Technology, Cambridge, MA, United States

**Keywords:** anosognosia, Alzheimer’s disease, error-monitoring system, emotional processing, neural mechanism, synaptic failure, error-related potentials, self-awareness

## Abstract

Although neurocognitive models have been proposed to explain anosognosia in Alzheimer’s disease (AD), the neural cascade responsible for its origin in the human brain remains unknown. Here, we build on a mechanistic dual-path hypothesis that brings error-monitoring and emotional processing systems as key elements for self-awareness, with distinct impacts on the emergence of anosognosia in AD. Proceeding from the notion of anosognosia as a dimensional syndrome, varying between a lack of concern about one’s own deficits (i.e., anosodiaphoria) and a complete lack of awareness of deficits, our hypothesis states that (i) unawareness of deficits would result from primary damage to the error-monitoring system, whereas (ii) anosodiaphoria would more likely result from an imbalance between emotional processing and error-monitoring. In the first case, a synaptic failure in the error-monitoring system, in which the anterior and posterior cingulate cortices play a major role, would have a negative impact on error (or deficits) awareness, preventing patients from becoming aware of their condition. In the second case, an impairment in the emotional processing system, in which the amygdala and the orbitofrontal cortex play a major role, would prevent patients from monitoring the internal milieu for relevant errors (or deficits) and assigning appropriate value to them, thus biasing their impact on the error-monitoring system. Our hypothesis stems on two scientific premises. One comes from preliminary results in AD patients showing a synaptic failure in the error-monitoring system along with a decline of awareness for cognitive difficulties at the time of diagnosis. Another comes from the somatic marker hypothesis, which proposes that emotional signals are critical to adaptive behavior. Further exploration of these premises will be of great interest to illuminate the foundations of self-awareness and improve our knowledge of the underlying paths of anosognosia in AD and other brain disorders.

## 1. Introduction

Anosognosia – a term derived from the Greek: “a,” absence; “nosos,” disease; “gnosis,” knowledge – was introduced more than a century ago by the French neurologist Joseph Babinski to describe the lack of awareness of a motor deficit resulting from right hemisphere damage (1). Since then, accumulating evidence has shown that anosognosia may affect any type of brain deficits or loss of function (2–4). For instance, it is now well established that unawareness for memory deficits is frequent from the early stages of Alzheimer’s disease (AD) (5), with evidence that it may have a predictive value for worsening of cognition over the disease course (6–8). Moreover, anosognosia typically delays AD diagnosis and causes resistance to treatment and rehabilitation efforts (9, 10), increasing the burden of care (11).

Interestingly, sometimes patients appear aware of their deficits in explicit verbal reports, but show a lack of emotional concern about the deficits and act inappropriately given their condition. This behavior, called anosodiaphoria, has been related to anosognosia in a putative *continuum* that suggests a dimensional rather than a categorical syndrome, ranging from the lack of concern for the deficits (with implicit unawareness, probably depending on a pre-conscious mechanism) to the complete lack of awareness with explicit denial of the deficits (that is, an explicit unawareness) (12). Note, however, that AD patients may also have preserved emotional reactivity to failure (implicit awareness) despite reduced awareness of performance (explicit unawareness) (13). This *double dissociation* between implicit and explicit awareness suggests instead a *bidirectional unawareness continuum*, with two poles, each following its own path, which constitutes the strongest pillar of our *dual-path hypothesis*. Like anosognosia, anosodiaphoria seems to increase with AD progression (12). Importantly, apathy – a motivational disorder characterized by loss of initiative and lack of emotional reactivity – has been associated with unawareness in AD, but unlike depression, greater apathy seems to correlate with higher levels of anosognosia (4, 14, 15). Additionally, an association between unawareness of deficits and executive dysfunctions in AD has been observed (16–18). More recently, using different neuroimaging techniques, researchers have found metabolic changes, namely reduced glucose metabolism in the posterior cingulate cortex (PCC) and the hippocampus (19), and/or functional disconnections within cortical midline structures involved in self-referential processes, including the medial orbitofrontal cortex (OFC) and the PCC, as well as disconnections between these regions and the medial temporal lobe in prodromal AD patients with anosognosia for memory deficits (20). In other cognitive domains unrelated to memory, anosognosia of deficits has been associated with structural lesions in the anterior cingulate cortex (ACC) (21), a brain region also involved in self-referential processing (22). Nevertheless, though the generation of new hypotheses explaining anosognosia has progressed in recent decades, the neural mechanistic cascade responsible for the emergence of anosognosia in the human brain remains largely unknown.

The Cognitive Awareness Model (CAM) (23), probably the most influential model of anosognosia in AD, is based on a modular framework that accounts for multiple levels at which unawareness phenomena can be generated, including sensory levels and differentiated levels within a hierarchy of memory consolidation processes. Specifically, the CAM predicts that various dissociations can be found in the relationship between awareness and memory (2), which would result in three main types of anosognosia: (1) Mnemonic anosognosia, reflecting a deficit in the consolidation of new information in the personal data base, which is at the origin of the metaphorical “*Petrified Self*” concept (24, 25); (2) Executive anosognosia, reflecting an impairment in the mechanism that normally allows for comparison between the actual performance and the stored past information; and (3) Primary anosognosia, reflecting the inability of patients to update their knowledge on their own cognitive functioning due to a dysfunction in the metacognitive awareness system (MAS). Specifically, the MAS is anchored in a comparator system that operates in tandem with a personal data base containing semantic representations of the self. Later research integrated the role of emotional processes in the CAM (26), building upon earlier work (27). For more information on this model, see also Tagai et al. (28) and Lenzoni et al. (25).

Our hypothesis derives from a distinct perspective: the core of our rationale is that anosognosia in AD, rather than being modular or domain-specific, would emerge from a critical breakdown in the system responsible for error detection and awareness, not necessarily tied to declarative memory processes (as in the case of the MAS). These errors could be committed in the context of any type of deficits, depending on their level of impairment. Specifically, we predict that AD patients *with* anosognosia would have a synaptic failure in the error-monitoring system, while AD patients *without* anosognosia would have this system still intact or, at least, able to compensate for a possible dysfunction through a “more firing, less wiring” mechanism (29) as part of a synaptic plasticity process (30, 31). In particular, this concept refers to the ability of synapses to modify their structure and/or function after persistent electrical activity, which seems the primary mechanism for learning and memory formation (32). Moreover, synaptic failure, resulting from alterations in intrinsic molecular mechanisms or from changes in biochemical reactions in the surrounding neural environment, is a common feature of several neurological and psychiatric disorders (33). Notably, it is one of the earliest pathological events to occur in AD (34–36), even before neuronal loss (37). This is of utmost relevance because it might be at the origin of cognitive deficits (38) and behavioral phenomena, like anosognosia (5), which characterize the prodromal stage of this devastating disorder. As such, cognitive event-related potentials (ERPs) may constitute a valuable biomarker for studying these clinical manifestations (39).

Hence, anosognosia would emerge from a necessary synaptic failure in the error-monitoring system, affecting patients’ awareness for any type of deficits, according to their proneness to produce errors in the concerned domains, as a consequence of (1) *direct* or (2) *indirect* damage to that system, as detailed below.

## 2. The dual-path hypothesis for the emergence of anosognosia in AD

The *dual-path hypothesis* predicts that the lack of awareness, with explicit denial of deficits (i.e., anosognosia, or explicit unawareness; *first case*) would result from *direct* damage to the error-monitoring system; whereas the lack of concern of deficits (i.e., anosodiaphoria, or implicit unawareness; *second case*) would more likely result from a disturbance in the emotional processing system, with *indirect* impact on error-monitoring. These (1) *direct* and/or (2) *indirect paths* would be at the origin of a neural mechanistic cascade leading to a critical synaptic failure in the error-monitoring system, and would eventually result in the inability of subjects to perceive (explicitly and/or implicitly) their own deficits in any domain (i.e., cognitive, behavioral, functional, etc.).

### 2.1. Three mechanistic predictions

**Figure 1:**
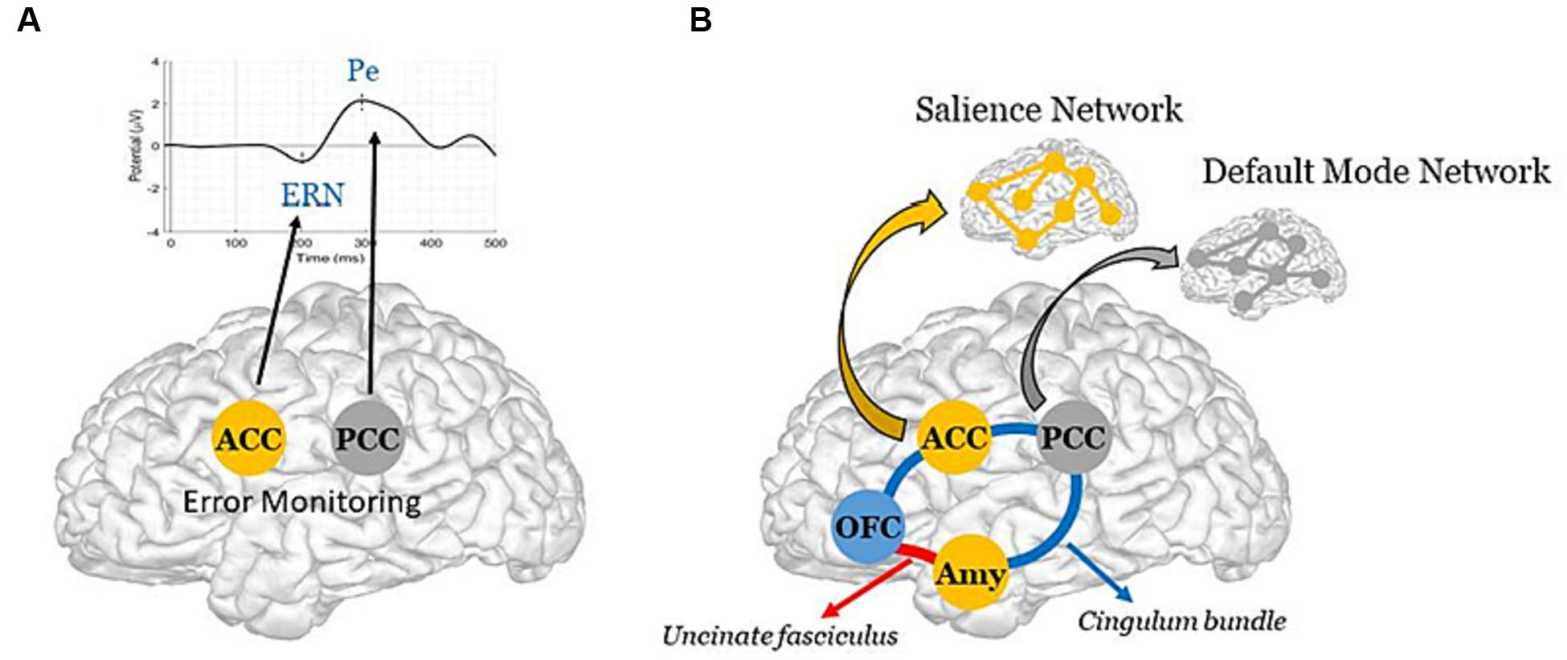
Schematic representation of the *Dual-path hypothesis for the emergence of anosognosia in AD*. (**A**, *Direct path*) Synaptic failure in the error-monitoring system, due to direct damage to the ACC and/or the PCC, the brain generators of the ERN and the Pe (40), with negative impact on explicit awareness. Note, reduced metabolism has been reported in the PCC from the early stages of AD (41), with further evidence of accentuated hypometabolism in AD patients whose the onset of symptoms was before 65 years old (42). (**B**, *Indirect path*) Structural and/or functional disconnections between the emotional processing and the error-monitoring systems, with negative impact on implicit awareness. The schematic diagram illustrates the cingulate fibers that travel along the anterior–posterior axis of the *cingulum bundle* (in blue) to reach various brain regions, including the medial temporal lobe, the Amy, and the OFC (43). The Amy and OFC are further interconnected via the *uncinate fasciculus* (in red) (44). Note, these two major white matter tracts have been shown to be impaired in the early stage of Alzheimer’s disease (45). Further illustrated are the ACC and the Amy, which constitute key brain areas of the SN; and the PCC, which constitutes a key region of the DMN [see Menon (46) for literature review on large-scale brain networks in psychopathology, highlighting potential parallels across neurological and psychiatric disorders].

Specifically, our *mechanistic predictions* are the following:

In the *first case*, a failure in the error-monitoring system would have a *direct* impact on error (or deficit) awareness, thus preventing patients from becoming aware of their condition. Such a failure would reflect local damage to the cingulate cortex, with particular focus on its anterior (ACC) and posterior (PCC) parts, which are the brain generators of ERPs associated with error monitoring, namely the error-related negativity (ERN) for preconscious error detection, and the positivity error (Pe) for error awareness (40) (Figure 1A). It is worth noting that, in AD, the interaction of oligomeric amyloid-β or misfolded tau protein with cell surface receptors might lead to changes in membrane/ion channel activity from its very early stages. This, in turn, could trigger a deterioration in synaptic structure and/or function with negative impact on network connectivity and information processing (47, 48). Remarkably, there is evidence of amyloid-β accumulation (49) and reduced number of synapses (50) in the PCC from the earliest stages of this neurodegenerative disorder. Yet, even in the case of a failure in the error-monitoring system, a preserved emotional processing system would assure some implicit awareness, with possible benefits on implicit learning and behavioral adjustment. In line with this view, recent evidence has found some implicit recognition of difficulties in AD patients despite their inability to explicitly estimate their own cognitive functioning (51).
In the *second case*, an impairment in the emotional processing system, in which the amygdala (Amy) and OFC play a major role (52, 53), would have an *indirect* impact on error-monitoring by rendering patients unable to detect relevant errors (or deficits) in the internal milieu, and to assign appropriate value to them. More precisely, such an impairment would bias the impact of deficits on the error-monitoring system, thus possibly affecting the generation of ERPs, and particularly the ERN, a preconscious error detection biomarker, whose amplitude appears modulated by motivational and emotional factors (54). Patients would therefore suffer from an implicit rather than explicit unawareness, being able to identify their own deficits though unable to understand their consequences and adapt their behavior. As such, localized impairment to the Amy and the OFC, along with potential structural and functional disconnections between these regions and critical nodes of the error-monitoring system, could underlie this implicit unawareness. In particular, these disconnections could result from (i) impairments in the integrity of two critical white matter pathways — the *uncinate fasciculus* (54, 55), which connects the OFC (a central hub for multisensory integration and the generation of somatic markers based on secondary emotions, especially in its medial part and in the adjacent ventromedial prefrontal cortex) to the Amy (which generates somatic markers from primary emotions, particularly involved in emotional arousal processes); and the *cingulate bundle* (56), an intricate network containing both lengthy fibers linking the cingulate cortex (encompassing the ACC and PCC, integral to error-monitoring); and/or (ii) a functional imbalance within and between two resting state brain networks, namely the *Salience Network* (SN) (55), anchored in the ACC, the Amy and other limbic structures, critically involved in the detection and response to relevant stimuli, and the *Default Mode Network* (DMN) (56), whose brain core is the PCC (57), principally involved in self-referential processes (58) (Figure 1B). Remarkably, the bases of impaired self-awareness and anosognosia have been closely linked to DMN functioning in AD (59, 60). In addition to the PCC, other DMN regions include the precuneus, medial prefrontal cortex, and bilateral temporoparietal junction (61). Interestingly, structural-functional connectivity impairments between the precuneus and the PCC, but also beyond the cingulate cortex, seem to play a central role in modeling AD as well as other neurodegenerative and psychiatric disorders (62). We further hypothesize that these disruptions may in part, albeit indirectly, impact the error-monitoring system.
A *third prediction*, following a synergistic A plus B mechanism (Figures 1A,B), would result in the most severe case of anosognosia, affecting both explicit and implicit awareness, with pronounced consequences, up to and including a full inability of patients to learn from their errors (or deficits) and to adapt their behavior.

## 3. Scientific premises and evaluation of the hypothesis

### 3.1. Two scientific premises

Our hypothesis stems on *two scientific premises*: the *one* comes from our preliminary results on ERPs showing a synaptic failure in the error-monitoring system of AD patients at the time of their diagnosis (63). In particular, by studying ERPs elicited by errors during a word memory recognition task in two groups of cognitively normal amyloid-positive individuals at baseline, we have recently shown direct evidence of an error-monitoring failure, along with a cognitive awareness decline, only in subjects who progressed to AD during the five-year study period. Specifically, we measured the ERN, mainly related to error detection, and the Pe, mainly related to error awareness, in 51 amyloid positive individuals who presented only subjective memory complaints at study entry, while they performed a word memory recognition task. Of these, 15 individuals progressed to AD within the five-year study period (PROG group), and 36 remained cognitively normal (CTRL group). We observed opposite longitudinal effects for the PROG and CTRL groups. Notably, we found a reduction of the Pe amplitude for the PROG group over time, in contrast with the increase of the Pe amplitude for the CTRL group. Moreover, contrary to the CTRL group, subjects who progressed to AD showed a cognitive awareness decline, with signs of anosognosia for their cognitive deficits at the moment of AD diagnosis (63). Importantly, there is evidence that error-monitoring impairments in AD patients, and consequent unawareness of errors (including errors committed as a consequence of memory recognition deficits), are not merely a byproduct of their typical memory impairment (64).

The *second* premise comes from the “somatic marker hypothesis,” proposed by Damasio (65, 66). In fact, our rationale converges to some extent with that hypothesis, which states that one must “feel” the consequences of one’s own actions, assigning them an affective value, in order to make the right decisions. In particular, Damasio proposes that decision-making (like self-awareness in the case of our *dual-path hypothesis*) requires the interplay between two specific brain systems: the executive and the emotional processing systems, in which the OFC (67) and particularly the amygdala (68) are necessary for triggering somatic states (53). Specifically, Damasio argues that autonomic reactions, such as electrodermal responses (EDR; see Sequeira et al. (69) for literature review on electrical autonomic correlates of emotion) to stimuli, might prepare the subject to adapt attentionally and physically to changes in the environment (66). Other researchers have also pointed to an important role for emotional dysregulation in producing unawareness, as errors may require an affective signature to motivate self-monitoring (27). Supporting evidence exists for severe AD pathology in autonomic-related cortices, such as the OFC, which suggests that it could contribute to the emotional and autonomic dysregulations that often accompany this neurodegenerative disorder (70).

In line with these premises, anosognosia of memory deficits has been associated with either hypoperfusion or hypometabolism in the PCC (7, 20, 71), the ACC (71), and the OFC (20, 71). Congruently, additional research has shown reduced within- and between-network connectivity in the DMN in AD patients with anosognosia (60, 72), with a pertinent association between hypometabolism in this network and an increased risk of progression to dementia in anosognosic patients (73).

Interestingly, evidence from another study found an association between memory monitoring and motor monitoring in AD patients, but observed that anosognosia for memory deficits was associated only with memory monitoring, not motor monitoring (74). The authors interpreted their results within a hierarchical model of awareness, suggesting that local self-monitoring processes (based on domain specific monitors) were associated across different domains, but only contributed to overall levels of awareness in a domain-specific manner. We interpret their results in a new light. Our rationale proposes that anosognosia is not domain-specific and can occur in any domain that is conducive to error. In early stages of AD, with a typical amnestic presentation, a dysfunction in error-monitoring would largely concern memory deficits, rather than other less affected or even unaffected functions, explaining why overall unawareness (or anosognosia for memory deficits as assessed through an offline, clinical interview method in that study) would be related to a failure in memory monitoring, but not to motor monitoring. Or, to put it another way, we postulate that if there were no errors, there would be no anosognosia. Therefore, a failure in the error-monitoring system would explain why anosognosia tends to worsen during the course of AD, following the level of impairment (and, consequently, the probability of making errors) in various cognitive, behavioral and functional domains, beyond memory.

### 3.2. Evaluation of the hypothesis

A new line of research is thus needed to explore the *dual-path hypothesis for the emergence of anosognosia*. We aim at investigating this hypothesis by studying a group of AD patients, at distinct stages of the disease, presenting different levels of anosognosia, versus a group of healthy elderly controls, while performing a computer-based error-monitoring task. To characterize our study population, we will use a comprehensive neuropsychological battery, including several cognitive tests to assess global cognition, memory, executive and instrumental functions, as well as both *online* performance discrepancy measures (based on our computerized task) and the *offline* Healthy Aging Brain Care Monitor questionnaire to detect anosognosia in distinct cognitive, psychological/behavioral and functional domains (63, 75, 76). In addition, standard scales will be used to evaluate depression, anxiety and apathy, which is highly relevant because these neuropsychiatric conditions can have an impact on the amplitude of our electrophysiological biomarkers of interest (54, 77, 78), and so must be taken into account in the statistical models. Specifically, we will focus our research on the study of erroneous responses and their possible correlations with the level of anosognosia and both central (i.e., the ERN and the Pe) and peripheral (i.e., the EDR) biomarkers of error awareness and emotional arousal, measured simultaneously during the computer-based task. Because of their extremely high temporal resolution, cognitive ERPs, such as ERN and Pe, are a valuable, non-invasive tool for assessment of synaptic dysfunction (39, 79). Also, we will investigate to what extent the level of anosognosia can be correlated with the amplitudes of these electrophysiological biomarkers, as well as with our hypothesis’ main regions (as illustrated in Figure 1: the PCC, the ACC, the OFC and the Amy), which have been associated with neural mechanisms of autonomic, emotional, and cognitive integration (67, 68, 80), and their interconnections, through structural and functional neuroimaging methods.

## 4. Discussion

While new hypotheses have been put forward over the last decades to explain anosognosia, there is still no evidence of a neural mechanistic understanding of this phenomenon. Here, we hypothesize that anosognosia might emerge from a critical synaptic failure in the error-monitoring system, thus preventing patients from detecting (explicitly and/or implicitly) their own errors (or deficits). This failure could result from either (i) direct damage to the error-monitoring system (i.e., *Direct path*, Figure 1A) or from (ii) the lack of emotional feedback on errors arriving to that system (i.e., *Indirect path*, Figure 1B), reflecting, in this case, local impairment in key structures of the emotional processing system and/or a disconnection between this system and the error-monitoring system.

We have focused our hypothesis on AD as a pathological model, but our rationale implies that it can be applied to any other brain condition in which anosognosia occurs. Indeed, anosognosia is prone to main deficits, according to their level of impairment, in several brain disorders. As it is well known, in chronic, progressive disorders, like AD, anosognosia tends to worsen over the course of the disease. We interpret this association in a particular way. We propose that anosognosia probably follows the appearance of errors committed in the context of a given deficit, such as deficits in episodic memory and the consequent forgetfulness (that is, the error), which generally occur from the early stages in AD patients. Since errors would become more frequent as the disease progresses, the level of anosognosia would correspondingly increase. On the contrary, in non-progressive disorders such as acute stroke, anosognosia is often a transient phenomenon, probably benefiting from a cascade of mechanisms leading to synaptic plasticity as frequently observed within the first hours after the onset of cerebral ischemia (81). To provide a few examples, anosognosia is mostly related to episodic memory deficits in the early stages of AD, but it can also affect other deficits following the severity of the disease (82); as it is also mostly related to personality changes in the behavioral variant of frontotemporal dementia (83); or to hemiplegia, particularly in the acute phase after a right-hemisphere stroke (84); etc. Altogether, this strongly suggests that a common inability to monitor errors (committed in the context of a given deficit), more than a specific memory consolidation impairment typical of AD, may be at the origin of this intriguing syndrome. Importantly, this would hold true regardless of the type of deficits (cognitive, behavioral, motor, etc.) or the neurological condition (neurodegenerative disorder, stroke, etc.).

For instance, in the context of stroke, there is evidence of action-monitoring deficits in patients with anosognosia for hemiplegia (AHP) (85, 86). Interestingly, these action-monitoring deficits seem to relate to monitoring deficits in distinct cognitive domains (87), thus supporting the existence of an error-monitoring impairment in stroke patients with AHP. Moreover, these patients appear unable to monitor self-performed actions, while able to monitor others’ actions or their own actions as if they were a third person (88), which seems to indicate impairment in their self-referential systems. As clearly established, the DMN – and the PCC, as a central node of this network – play a primordial role in self-referential processes, with additional evidence indicating that damage to brain white matter tracts involved in these processes may foster the appearance of anosognosia in stroke patients (89).

A growing body of evidence has shown that synaptic failure is a common pathological finding in several brain conditions, including neurodevelopmental and neurodegenerative diseases (33), as well as ischemic cerebral damage. Interestingly, although not focused on anosognosia, further evidence has suggested that prolonged synaptic failure may be a cause of persistent symptoms in patients with cerebral ischemia (90). Moving research from correlation to understanding the mechanistic causation is crucial for the development of successful therapies in the neurological and psychiatric fields. ERPs primarily reflect synaptic transmission processes, and may thus provide sensitive biomarkers to improve our knowledge on the neural substrates and mechanisms underlying brain disorders and their clinical manifestations.

Finally, elucidating the neural mechanistic cascade leading to anosognosia in AD may have two major clinical and scientific outcomes: *first*, contribute to a deeper understanding of the pathophysiology of this neurodegenerative disorder; *second*, refine current models of anosognosia with the goal of improving rehabilitation strategies allowing anosognosic patients to adhere to healthcare measures, which could maintain their autonomy for longer and reduce the burden of care. Very important, the rationale of our hypothesis extends beyond AD. To validate it, novel research is required not only in Alzheimer’s patients, but also in other neurological (and even psychiatric) populations, in which anosognosia has been frequently reported. Such a line of research can still illuminate the theoretical foundations of human self-awareness.

## Data availability statement

The original contributions presented in the study are included in the article/supplementary material, further inquiries can be directed to the corresponding author.

## Author contributions

KA: conception of the hypothesis, manuscript writing (first draft and final version). DP: manuscript review and editing, design of the “hypothesis figure,” conceptualized with KA, with further contributions of TG and TM. All authors: discussion of arguments in support of the hypothesis.
